# Child-Nature

**Published:** 1871-10

**Authors:** P. H. Hayes


### CHILD-NATURE.

P. H. HAYES, M. D.

What is sweeter than the love and faith of children? What this side of heaven so heaven-like as uncorrupt child-nature? What dream of heaven so pure as that which comes to the heart of a child?

"I hold it a religious duty,
To love and worship children's beauty;
They've least the taint of earthly clod,
They're freshest from the hand of God."

There is not to me a sadder thing in all the world, than to see many even good parents unconsciously at war with the child-nature of their own children; they have forgotten their own child-days, child-thoughts, child-enjoyments, child-fears, and child-like mistakes.

Little Jennie, who is but six years old, passed her plate for toast and warmed-up potato at the same time, but when the toast gravy flowed across her plate and mixed with the potato, she was shocked. To her child-thought it was a grievous accident. Her little chest swelled, and tears began to blind her eyes. Her mother said, "No matter, Jennie, it's just as good." Jennie cried, the mother chided, and my heart took sides with Jennie. I thought of the great distance of age and experience between that mother and her child; and how rarely we see parents who can return over the distance between them and childhood, and enter by true love and sympathy into the whole nature and needs of children. Even the chiefest of the Apostles, whose teachings take hold on all the grand questions concerning this life and the next, remembered his childhood when he said, "When I was a child I spoke as a child, I understood as a child, I thought as a child." Verily, how often is our temper ruffled with children, because as children they think and talk and act. We might as well find fault with them for being born into childhood at all.

A little boy came home from his fishing with a few minnows, about as large as his fingers in truth, but, measured by his own eyes, big fish indeed. I heard him, all out of breath, trying to tell his mother where he caught this one, and how that one, and would she have Celia cook them all for his supper? Oh! mother of that pure type of boy-nature, why did you not let your heart down—no, I should say *up*—into sympathy with your boy's success, of which he was so proud? But you looked down at his boots and then at his pantaloons, and then you exclaimed, "Why, Robbie! where *did* you find all that *mud!*"

Little Ella G—— is fairly imprisoned in her fine clothes. She cannot go out and play with the abandon of real childhood, she is deprived of that very child-like freedom which is the instinct and demand of nature, and absolutely necessary for health, growth and development. Her mother has fairly beaten into her a sort of womanly care and caution about her dress, witting not that herein lies the whole gist of the reason why she has to call the doctor so often for Ella, and say of her, "She is such a *delicate* and *nervous* child."

A little fellow called out in the night, "Father! father! are you there?" The father answered, but scolded his boy and told him he must not wake him up for nothing. The next night he called again, "Father! I'm hot, come and take off the clothes." The father went and found his boy in a state of feverish wakefulness. This time he won his boy's heart by tenderness, and the little fellow told him that he had read all about the murder of Mr. Nathan in the paper, and he waked up and got to thinking, what if some awful wicked man should come to kill his father in the dark night.

Albert D—— is eleven years old. For a long time his uncle had been in the habit of showing him his watch and ending by saying, "Some day I shall give it to you." Not long ago this uncle died, and in his will he gave this watch to Albert. This Albert knew, and how could he help thinking about it almost every hour in the day. A watch—a man's watch—all his own! Time passed; the uncle's things were all left in his room as they were when he died. Albert grew impatient and at last thought within himself, "The watch is mine; what harm in my putting it on for an hour?" So he stole into the room, put on the watch, and ran down stairs and out into the snow to show his watch to his school fellows and have a good time. But judge, if you can, ye who are grown and stout-hearted, of the sorrow of this boy when, as he was about to return into the house he discovered that he had lost the watch in the snow. And now to add to his calamity, what would his father and mother say—what would they do? Is it possible so to train children that in just such extremities as this they shall have the courage to hasten to their parents for the luxury of confession and forgiveness—a "perfect love which casteth out fear."

But Albert did not dare approach his parents with his dread secret, nor did they, when they missed the watch and suspected Albert had taken it, see this golden opportunity to be magnanimous to their already doubly punished boy. Why did they not take him to some secret chamber, quiet his fears, and win from him by love his heart-breaking secret. But no, they stormed and scolded, and he, terrified, denied his guilt, cried, and persisted in his denial for a whole day, and then, wearied out, confessed all. Weeks after this I saw this boy, and he looked as though reproach had broken his heart, and he had retired to live alone in the solitude of his own soul.

The tyrrany of *fear* represses child-feeling and starves the need of the child-heart for love. It is *love* only can develope the *good* in us. It is only a few years ago that a quiet "Greenwood" grave received all that could die of an "erratic genius, an accomplished, eloquent actress and speaker, a warm-hearted and impulsive woman, a reckless, sorrowing, penitent, redeemed magdalen." It was the bitter child-life of this nature's noble woman that warped and blotted her after-life. Here is a bit of her own record of herself, which, spoken as it was when she was drawing crowds to her ballet dances, and causing the cheek of modesty to burn, sounds like sighs from the depths of a harlequin's hell: "Happy? *I* happy? No. I never had a truly happy day in all my life. Restless? bitten by the Spanish spider? Yes, indeed, bitten by despair. My mother never *loved* me; she turned me from her breast to a hired nurse. I was alien from babyhood. I was only six years old when she sent me from home to a hard Scotch school. I tell you my life has been barren of much good. I never had a *real mother*, a *real home*, a *real love*."

